# Association between Thai language proficiency and adherence to COVID-19 protective behaviors (CPB) among Myanmar migrant workers in Southern Thailand

**DOI:** 10.1371/journal.pone.0312571

**Published:** 2024-10-25

**Authors:** Hein Htet, Aungkana Chuaychai, Tida Sottiyotin, Kyaw Ko Ko Htet, Hutcha Sriplung, Wit Wichaidit, Virasakdi Chongsuvivatwong

**Affiliations:** 1 Department of Epidemiology, Faculty of Medicine, Prince of Songkla University, Hat Yai, Songkhla Province, Thailand; 2 Department of Preventive and Social Medicine, Ministry of Health, University of Medicine (Taunggyi), Taunggyi, Myanmar; 3 Department of Pharmaceutical Care, School of Pharmacy, Walailak University, Nakhon Si, Thammarat Province, Thailand; 4 Drug and Cosmetics Excellence Center, Walailak University, Nakhon Si, Thammarat Province, Thailand; Thammasat University Institute of East Asian Studies, THAILAND

## Abstract

The association between host country language proficiency and disease prevention among migrants is underexplored. The objective of this study is to assess the extent to which self-reported command of the Thai language is associated with adherence to COVID-19 protective behaviors (CPB) among Myanmar migrant workers in Thailand. We distributed a self-administered structured questionnaire in Burmese language to 1,050 Myanmar migrant workers in Southern Thailand from September 2022 to January 2023. The questionnaire included background characteristics, self-reported Thai language proficiency based on the Common European Framework Reference (CEFR), and self-reported CPB adherence at residence and workplace. We analyzed data using descriptive statistics and multivariate linear regression analysis. Although slightly less than half of the participants reported CEFR A1 level or higher in Thai speaking and listening skills, less than 10 percent did so for reading and writing skills. Workplace COVID-19 preventive adherence scores were initially found to be significantly associated with A1 level or higher speaking and listening skills. However, after adjusting for confounders, these associations were not statistically significant (Speaking skill’s Adjusted Beta = 0.713, 95% CI = -0.011, 1.437; Listening skill’s Adjusted Beta = -0.367, 95% CI = -1.087, 0.353). No significant associations were found between any language skill domain and residence COVID-19 preventive adherence scores for both unadjusted and adjusted analysis. The study findings may have implications for relevant stakeholders in migrant services, migrant health, and infectious disease control. However, information biases, language barriers, and lack of generalizability should be considered as caveats in the interpretation of the study findings.

## Introduction

Transnational migrants play a crucial role in Thailand’s socio-economic development, representing over 10% of the total labor force [1]. Nationals from Myanmar constitute 68% of the migrant workforce in Thailand [1]. Migrant workers were particularly vulnerable during the COVID-19 pandemic in Thailand, with a high number of infections [2–6], particularly among those from Myanmar [7]. Migrant workers tend to live in overcrowded accommodations with insufficient protective measures, which can contribute to outbreaks of COVID-19 beyond the migrant community [8]. For example, asymptomatic infections among Myanmar migrant workers in Samut Sakhon Province triggered the second wave of COVID-19 pandemic in Thailand [9].

A study reported that only one-third of Myanmar migrant workers in southern Thailand regularly adhered to COVID-19 preventive measures, including mask-wearing, hand-washing, and physical distancing during the pandemic [10]. During the COVID-19 pandemic, a survey by the International Labour Organization reported that 57% of migrant workers in Thailand lacked facemasks and hand sanitizers [11]. The International Organization of Migration also reported that Myanmar migrants were in need of employment, food assistance, hygiene items, medicines, medical support, and personal protective equipment [12, 13]. The Migrant Working Group reported that migrant workers needed protective equipment and dried foods and faced challenges in implementing distancing measures due to overcrowded, unsanitary living conditions [14].

The World Health Organization reported that migrants often face significant obstacles in accessing health services in their host country, including language and communication barriers [15]. In the field of public health, language is often identified as a major healthcare barrier for migrants in Thailand [16] and other host countries [17]. Previous studies among migrant populations in Western countries found that the host country’s language proficiency was positively associated with infectious disease prevention, screening, and management [18–20].

In Thailand, migrant workers from Myanmar typically have low-skilled occupations [1], a low level of education [21–23], and a limited ability to communicate in Thai language [24, 25]. Although previous studies have shown that Thai language proficiency is associated with low COVID-19 vaccination [26] and a lack of COVID-19 healthcare information [27] among Myanmar migrant workers, several knowledge gaps still exist. Language proficiencies generally include four domains (speaking, listening, reading, and writing) [28], and a lower proficiency in one domain can be supplemented by higher proficiency in others. Thus, all of these domains need to be measured in other to accurately evaluate the extent to which language proficiencies are associated with COVID-19-related outcomes [28, 29]. However, previous studies tended to measure only speaking and listening skills among Myanmar migrants [25], which provided an incomplete picture of language proficiency. Migrants with moderate speaking skills who could read and write Thai would arguably be more capable of independently accessing Thailand’s healthcare system. A related issue was the measurement of language competency [29], which varies widely among different migrant populations [30–33] without standardization and may involve resources at a level not suitable for rapid population surveys.

Utilizing the *Common European Framework of Reference* (CEFR) [29], a globally recognized standard guideline for measuring foreign language ability, could enable a more comprehensive assessment of self-reported language proficiency. The CEFR provides a standardized framework for assessing foreign language proficiency comprising six levels, namely A1, A2, B1, B2, C1, and C2, each with an increasing level of proficiency [34]. The A1 level designates the ability to understand and use familiar everyday expressions, introduce oneself, and make simple interactions with users of the language [35]. The B1 level refers to the ability to understand the main points of conversations on familiar matters, produce simple texts on familiar topics, describe experiences and ideas, and explain opinions and plans [35]. The C1 level refers to the ability to understand complex clauses with implicit meaning, use the language for all purposes flexibly and effectively, and produce texts on complex subjects [35]. Adapting the CEFR’s definition for self-reported assessment can offer rapid measurement of language proficiency in all four domains and help us assess the extent to which language proficiency is associated with infectious disease prevention behaviors among vulnerable migrant populations. Stakeholders in migrant health and health promotion can use these study findings as baseline information on the extent to which the target population understand the host country’s language, and the extent to which such understanding is associated with COVID-19 protective behaviors. Similarly, stakeholders in infectious disease prevention and control can use the findings to inform and justify the need to provide interpreters and other language services, or lack thereof, when working with language-diverse populations. The objective of our study is to assess the extent to which self-reported command of the Thai language according to the CEFR is associated with adherence to COVID-19 protective behaviors among Myanmar migrant workers in Thailand.

## Methods

### Study design and setting

We conducted a community-based cross-sectional study in Southern Thailand (Hat Yai City Municipality and Pattani City Municipality), urban areas with various factories, construction sites [36, 37], and fisheries [21]. We collected data from 1 September 2022 to 24 January 2023, a period marked by a decline in COVID-19 cases in the local areas [38].

### Study participants

We recruited migrant workers from factories, construction sites, and fisheries in the study area. Inclusion criteria were: i) Migrant from Myanmar; ii) Age at least 18 years old; iii) Have lived and worked within the study area for a minimum of six months, and iv) Able to communicate in Burmese language. Exclusion criteria included: i) Having mental or auditory impairments, and ii) History of receiving COVID-19 vaccination outside of Thailand (to ensure that the findings pertained only to the Thailand context). The sample size for our analysis was calculated for a broader cross-sectional study with an objective to describe COVID-prevention behaviors among migrants from Myanmar.

We calculated the sample size using an infinite population proportion formula with adjustment for the design effect:

$$n=\frac{Z_{\left(1-a/2\right)}^{2}*p(1-p)}{d^{2}};\;n_{adjust}=n*deff$$

We assumed that 50% of migrants would adhere well to CPB (p = 0.50), the margin of error of 5% (d = 0.05), the confidence level of 95% (Z_(1-a/2)_ = Z _(1–0.05/2)_ = Z _(0.975)_ = 1.96, the design effect of 2 (deff = 2), the final sample size of migrants was determined to be 1,050 participants after accounting for the 20% non-response rate. Details about the sampling process and sample size calculation are outlined in a previous publication [39].

### Study variables

#### Exposure variable: Thai language proficiency

We measured Thai language proficiency based on the CEFR Framework question guidelines [34, 40]. The investigators considered the guidelines, deliberated regarding skills that corresponded to a given CEFR proficiency level within the migrants’ contexts. We designed our questions for self-reported proficiency up to the B1 level. There were three questions for each of the four skills: listening, speaking, reading, and writing (i.e., 12 questions in total). Each question had three possible answers: 1) “Can’t do”; 2) Partly do”, and 3) “Completely do”. We translated the question from English to Burmese but did not perform back-translation and revisions due to time and resource constraints. We tried to ensure the clarity and contextual relevance of the initial translation by asking native speakers of Burmese with subject matter expertise to check the translated question. We then classified each language proficiency skill into six categories as: "none," “less than A1 level,” “A1 level (beginner),” “A2 level (elementary),” “B1 level (intermediate),” and “incoherent response”. We have included details of our classification method in S2 Table. We reclassified incoherent responses based on the notion that when discrepancies occur in the self-reporting of language proficiency, the participant’s actual proficiency would be at the middle between the two discrepant levels. For example, those reporting ability to engage in speaking activities at A1 and B1 levels, but not at A2 level would be classified as having A2 level of speaking ability.

#### Outcome variable: Adherence to COVID-19 protective behaviors

Adherence to CPB refers to the extent to which an individual is likely to comply with COVID-19 preventive recommendations across a range of health behaviors, including hand washing, social distancing, and mask wearing, etc. The research team developed an eight-item tool for assessing workplace adherence to CPB, as well as a nine-item tool for assessing adherence to CPB in residential areas. The research team adapted these questions from the COVID-19 preventive guidelines based on information from the World Health Organization [41–44], the US Center for Disease Control (CDC) [45], Thailand’s Ministry of Public Health [46, 47], and a previous study [48]. Participants responded to the question items with 5-point Likert scale answer options: “*Rarely*”, “*Occasionally*”, “*Commonly*”, “*Mostly*”, and “*Always*”. We collapsed categories and classified participants as either those who “*Mostly*” or “*Always*” engaged in a given behavior (with a value of 1 in data analyses) or those otherwise (with a value of 0 in data analyses). We decided to collapse the categories because we deemed our Likert scale to be ordinal but subjectively interpreted (i.e., there was no measurable distance between "*Mostly*" and "*Always*"; each participant would have their own subjective interpretation. However, we also deemed “*Mostly*” and “*Always*” to refer to behavior occurring on more than half of all occasions, and that this notion was adequately different from “*Commonly*”, “*Occasionally*”, and “*Rarely*” that no substantial overlaps would have existed when we dichotomized the responses. We then calculated a composite adherence score for both the workplace and residence separately by summing up the individual question items.

Psychometric evaluation of adherence scales was performed by both exploratory and confirmatory factor analyses which was presented in our prior research work [39], revealing Cronbach’s alpha reliability scores of 0.852 for adherence at residence and 0.942 for adherence at the workplace. We found that three question items in residential-area CPB had poor factor loading scores in exploratory factor analysis (EFA) and poor model fit in confirmatory factor analysis (CFA). Thus, we removed these three residential-area CPB question items from the analyses, resulting in a final set of six items in residential-area CPB and eight items for workplace CPB being used to construct our outcome variable.

#### Background characteristics of the study participants

We also measured the participant’s background characteristics including demographic, socioeconomic, migration history, and documentation status. Demographic and socioeconomic characteristics included age, sex, ethnicity, religion, education, marital status, living status, occupation, and monthly income. Migration history included duration of stay in Thailand, frequency of changing jobs in the past 5 years in Thailand, and frequency of changing residence in the past 5 years in Thailand. Documentation status included possession of legal documents and health insurance coverage.

### Study instrument

The study instrument was a self-administered questionnaire. The investigators developed the instrument through internal discussions and consultation with experts in epidemiology and public health management. We did not undertake a systematic content validity assessment due to time and resource constraints. The instrument included seven parts: 1) socio-demographic characteristics (10 items); 2) electronic health literacy (8 items); 3) online health information seeking behavior (6 items); 4) Thai language proficiency skills (12 items); 5) NCD risk behaviors (4 items); 6) adherence to COVID-19 protective behaviors (17 items), and; 7) COVID-19 vaccination (2 items). Investigators drafted the questionnaire in English and translated the questionnaire to Burmese. The questionnaire was then back-translated from Burmese to English, except the Thai language proficiency skills section which was not yet finalized. The investigators then asked native speakers of Burmese in Hat Yai city to evaluate the clarity and comprehensibility of the translation and made revisions to the question items and wording accordingly. The investigators then pilot-tested the revised study instrument among 30 Myanmar migrant workers in Hat Yai city (not at the same location at the study sites) to finalize the study instrument.

### Data collection

After getting ethical approval, the research team sent the official request letters to factories, constructions, and fisheries in the study area, and appointments were made before the survey. For undocumented migrant workers, major sites of their workplaces and residences were identified first with the help of the local key informant. Based on this information, eligible migrant workers were approached, informed about the study, and invited to participate (more details in the Ethical Considerations sub-section). After getting the appointment dates and permissions, data were collected using the self-administered questionnaire, which took approximately 30–45 minutes. Before the survey, the data collector read and gave the participant information sheets to the migrants in the Burmese language, explained the purpose and voluntary nature of the study, procedures, potential risks and benefits, and clarified issues regarding confidentiality, anonymity, informed consent, and the importance of honesty in the response. We only obtained verbal consent from the participants due to the sensitive nature and to maintain the confidentiality of their immigration status. Among approximately 20 participants who were illiterate or otherwise unable to read the questionnaire or write the responses, the data collector conducted face-to-face interviews. The data collector verbally explained the interview procedures to the participants, reassured the participants regarding the anonymity of their responses, and strictly followed the questionnaire when administering the interview.

### Data management and analysis

Epidata version 3.1 was used for data entry and R software version 4.0.2 was used for data analysis. We summarized descriptive statistics for background socio-demographics, the level of self-reported Thai language proficiency skills, and adherence to CPB both at the workplace and the residence. Multivariate linear regression analyses were conducted to determine the association between the four Thai language proficiency skills (categorical variables) and adherence to CPB at the place of residence as well as the workplace (mean adherence scores for workplace and residence preventive behaviors used as two separate continuous outcome variables). We treated those with incoherent responses regarding language proficiency as missing and excluded from the analyses. Based on previous literature, we identified age, sex, income, education, and length of stay in Thailand as the confounders for the association between language proficiency and adherence to CPB, both at the workplace and the residence [18, 49, 50] and adjusted our multivariable linear regression models for these characteristics accordingly. We conducted all regression analyses at a 95% level of confidence. We also conducted diagnostic assessments of the multivariable regression models. The data set and the R codes necessary to replicate the findings in this study can be found in the Supporting Information section.

### Ethical considerations

We received ethical approval from the Human Research Ethics Committee of the Faculty of Medicine, Prince of Songkla University, Hat Yai, Thailand (REC.65-071-18-1), the study was conducted in full compliance with international guidelines for the protection of human research subjects and COVID-19 preventive guidelines. Before data collection, the data collector provided information to potential participants according to the participant information sheet (PIS) in the Burmese language, which explained the purpose and voluntary nature of the study. The data collector then clarified issues regarding confidentiality and anonymity. We requested and received the exemption of obtaining written informed consent from the Human Research Ethics Committee, and only obtained verbal informed consent due to the confidentiality and sensitivity issues associated with the participants’ immigration status. A copy of the approved information sheet and verbal consent script can be found in the Supplementary Material section.

## Results

We approached 1350 migrants, among whom 1050 agreed to participate (response = 77%). Most participants were males, Burmese, Buddhists, married, and factory workers (*Table 1*). Nearly one-third had a secondary level of education, with a median monthly personal income of 9500 Thai Baht (THB). Most respondents had lived in Thailand for less than five years, with their family or relatives. More than three-fourths of the respondents (79.1%) had neither migrated (those who did not move from one place or province to another) nor changed jobs (those who have not switched from one employer or occupation to another) within the past five years in Thailand. The majority of the respondents had health insurance and legal documents. More than half of the respondents had no Thai language proficiency [i.e. those who indicated that they had no ability to speak, understand, read, or write in Thai] in reading (93.0%), writing (93.5%), listening (52.7%), and speaking skills (52.6%), followed by A1 level in reading (3.0%), writing (4.3%), listening (38.3%), and speaking skills (35.3%).

**Table 1:** Background characteristics of the respondents (n = 1050).

Variable	Frequency (%)
**Age groups**	
18 to 30 years	411 (39.1%)
31 to 43 years	508 (48.4%)
44 to 60 years	131 (12.5%)
**Sex**	
Male	593 (56.4%)
Female	457 (43.5%)
**Ethnicity**	
Burmese	565 (53.8%)
Rakhine	206 (19.6%)
Mon	107 (10.2%)
Kayin	86 (8.2%)
Htawei	48 (4.6%)
Others*	10 (1%)
Missing	28 (2.7%)
**Religion**	
Buddhist	1007 (95.9%)
Others^+^	27 (2.6%)
Missing	16 (1.5%)
**Marital status**	
Married	629 (59.9%)
Single	377 (35.9%)
Divorced/widowed/separated	26 (2.5%)
Missing	18 (1.7%)
**Education**	
Less than Primary, illiterate	57 (5.4%)
Less than Primary, can Read and write	178 (17%)
Primary school	157 (15%)
Secondary school	364 (34.7%)
High School	207 (19.7%)
University	48 (4.6%)
Missing	39 (3.7%)
**Job**	
Construction	243 (23.1%)
Factory	669 (63.7%)
Seafarers	137 (13.1%)
Missing	1 (0.1%)
**Monthly personal income (Baht)** Median (IQR)	9500 (8000–10000)
**Living status**	
Alone	199 (19%)
With family & relatives	643 (61.2%)
With friends	207 (19.7%)
Missing	1 (0.1%)
**Duration of stay in Thailand**	
<5 years	558 (53.2%)
≥5 years	491 (46.8%)
**Frequency of migration in Thailand within 5 years**	
Never	831 (79.1%)
1 – 2 times	154 (14.7%)
3 times or more	65 (6.2%)
**Frequency of changing job in Thailand within 5 years**	
Never	831 (79.1%)
1 – 2 times	161 (15.3%)
3 times or more	58 (5.5%)
**Having health insurance** ^†^	
No	99 (9.4%)
Yes	951 (90.6%)
**Having Legal document** ^‡^	
No	68 (6.5%)
Yes	982 (93.5%)
**Thai language command as per CEFR levels description**	
**Speaking**	
None	552 (52.6%)
Less than A1 level	7 (0.7%)
A1 level (Beginner)	371 (35.3%)
A2 level (Elementary)	9 (0.9%)
B1 level (Intermediate)	33 (3.1%)
Incoherent response	78 (7.4%)
**Reading**	
None	976 (93.0%)
Less than A1 level	6 (0.6%)
A1 level (Beginner)	31 (3.0%)
A2 level (Elementary)	0 (0.0%)
B1 level (Intermediate)	3 (0.3%)
Incoherent response	34 (3.2%)
**Listening**	
None	553 (52.7%)
Less than A1 level	24 (2.3%)
A1 level (Beginner)	402 (38.3%)
A2 level (Elementary)	8 (0.8%)
B1 level (Intermediate)	27 (2.6%)
Incoherent response	36 (3.4%)
**Writing**	
None	982 (93.5%)
Less than A1 level	14 (1.3%)
A1 level (Beginner)	45 (4.3%)
A2 level (Elementary)	3 (0.3%)
B1 level (Intermediate)	3 (0.3%)
Incoherent response	3 (0.3%)
**Workplace COVID-19 protective behaviors (mostly or always performing the behavior in the past 7 days prior to the survey)**	
Wearing face mask	739 (70.4%)
Cleaning hands before or after touching eyes/nose/mouth	627 (59.7%)
Sanitizing hands with soap and water/ alcohol-based sanitizer	643 (61.2%)
Washing hands for at least 20 seconds	537 (51.1%)
Covering face with a handkerchief/ bent elbow while coughing/sneezing	567 (54.0%)
Sanitizing personal items (e.g., your table, your instruments) use in workplace	522 (49.7%)
Maintaining a minimum distance of 1 meter at workplace	596 (56.8%)
Maintaining a minimum distance of 1 meter while eating food with your colleagues at workplace	612 (58.3%)
**Workplace COVID-19 protective behaviors score,** Mean ± SD	4.6 ± 3.1
**Residence COVID-19 protective behaviors (mostly or always performing the behavior in the past 7 days prior to the survey)**	
Wearing face masks while going out of home	834 (79.4%)
Regular opening of windows in home to improve air flow	812 (77.3%)
Sanitizing hands with soap and water/ alcohol-based sanitizer after touching eyes/nose/mouth	661 (63.0%)
Covering face with a handkerchief/ bent elbow while coughing/sneezing	629 (59.9%)
Sanitizing frequently touched objects and surfaces in your home	526 (50.1%)
Washing clothes worn in workplace when back from work	871 (83.0%)
**Residence COVID-19 protective behaviors score,** Mean ± SD	4.1 ± 1.9

* Others included Chin, Kayan, Palaung, Pao, and Shan

^+^ Others included Christian, Hindu, and Islam

^#^ Underlying diseases included hypertension, asthma, diabetes, back pain, gastritis, abdominal pain, dizziness, dysuria, and heart disease

^†^ Health insurance through Social Security Scheme or Migrant Health Insurance Scheme

^‡^Legal document included passport, work permit, CI book, pink card, and Myanmar National ID card

Although unadjusted linear regression models (*Table 2*) showed that workplace COVID-19 prevention behaviors adherence scores were significantly associated with Thai speaking and listening skills. However, multivariate linear regression models showed that the associations were not statistically significant (Speaking skill’s adjusted beta = 0.713, 95% CI: -0.011, 1.437; Listening skill’s adjusted beta = -0.367, 95% CI: -1.087, 0.353). The place of residence COVID-19 prevention behavior adherence scores had no significant association with any domain of Thai language proficiency (*Table 3*).

**Table 2:** Association between Thai language proficiency and adherence to workplace COVID-19 prevention behaviors (n = 1050).

Language Skill Domain and Level	Workplace CPB adherence score (Mean ± SD)	Unadjusted Beta (95% CI)	Adjusted* Beta (95% CI)	P-value for independence of residuals and VIF
**Speaking**				
Less than A1 or none (n = 559)	4.377 ± 3.230	0 (Ref.)	0 (Ref.)	P = 0.000, VIF = 3.320
A1 and above (n = 413)	4.831 ± 3.017	**0.453 (0.053, 0.853)**	0.713 (-0.011, 1.437)	
**Reading**				P = 0.000, VIF = 2.019
Less than A1 or none (n = 982)	4.610 ± 3.139	0 (Ref.)	0 (Ref.)	
A1 and above (n = 34)	4.618 ± 3.162	0.007 (–1.066, 1.082)	-0.461 (-1.964, 1.043)	
**Listening**				P = 0.000, VIF = 3.251
Less than A1 or none (n = 577)	4.463 ± 3.249	0 (Ref.)	0 (Ref.)	
A1 and above (n = 437)	4.812 ± 2.983	**0.349 (–0.040, 0.739)**	-0.367 (-1.087, 0.353)	
**Writing**				P = 0.000, VIF = 2.079
Less than A1 or none (n = 996)	4.583 ± 3.145	0 (Ref.)	0 (Ref.)	
A1 and above (n = 51)	5.196 ± 3.020	0.612 (–0.271, 1.497)	-0.261 (-1.628, 1.107)	

*Adjusted for other domains of language skills, age, sex, income, education, length of stay in Thailand

**Table 3:** Association between Thai language proficiency and adherence to residence COVID-19 prevention behaviors (n = 1050).

Language Skill Domain and Level	Residence CPB adherence score (Mean ± SD)	Unadjusted Beta (95% CI)	Adjusted* Beta (95% CI)	P-value for independence of residuals and VIF
**Speaking**				P = 0.001, VIF = 3.319
Less than A1 or none (n = 559)	4.072 ± 2.019	0 (Ref.)	0 (Ref.)	
A1 and above (n = 413)	4.136 ± 1.922	0.064 (–0.187, 0.315)	0.025 (-0.443, 0.492)	
**Reading**				P = 0.001, VIF = 2.019
Less than A1 or none (n = 982)	4.122 ± 1.970	0 (Ref.)	0 (Ref.)	
A1 and above (n = 34)	4.147 ± 1.909	0.024 (–0.648, 0.698)	0.072 (-0.899, 1.043)	
**Listening**				P = 0.001, VIF = 3.251
Less than A1 or none (n = 577)	4.090 ± 2.023	0 (Ref.)	0 (Ref.)	
A1 and above (n = 437)	4.162 ± 1.877	0.072 (–0.040, 0.739)	-0.085 (-0.551, 0.379)	
**Writing**				P = 0.001, VIF = 2.079
Less than A1 or none (n = 996)	4.124 ± 1.963	0 (Ref.)	0 (Ref.)	
A1 and above (n = 51)	4.235 ± 1.882	0.612 (–0.171, 0.316)	-0.249 (-1.132, 0.634)	

*Adjusted for other domains of language skills, age, sex, income, education, length of stay in Thailand

## Discussion

In this cross-sectional study, we assessed the extent to which self-reported Thai language proficiency was associated with CPB adherence among Myanmar migrant workers in Southern Thailand. The findings revealed that slightly less than half of the participants reported CEFR A1 level or higher in Thai speaking (39.33%) and listening proficiencies (41.61%), and less than 10 percent did so for reading (3.24%) and writing skills (4.86%). Workplace COVID-19 prevention behavior adherence was associated with Thai speaking and listening proficiencies, but the associations were non-significant after adjusting for confounders. There was no association between Thai language proficiency and COVID-19 prevention behavior adherence at the place of residence. Our study findings provide basic information that is potentially useful for stakeholders in migrant services, migrant health, and infectious disease control.

Based on the CEFR framework, approximately half of our participants reported an A1 (beginner) level of speaking (53.23%) and listening abilities in Thai (54.95%), but literacy was very low for reading (3.24%) and writing (4.86%). Several issues should be considered in the interpretation of our study findings. Firstly, language skills in our study were all self-reported and subjected to various biases (e.g., social desirability, response acquiescence, and self-serving biases) [51, 52]. Future studies with adequate time and resources could consider using an abridged version of standardized language tests instead of self-reporting [30, 53]. Secondly, we measured general language proficiency rather than health-related language proficiency. Thus, there was an issue regarding construct validity. Our language proficiency measurement questions were on general language skills (e.g., ability to tell a story to the police when asking for help) and not on the language skills specific to navigating the public health system (e.g., ability to describe disease history and symptoms to healthcare providers). Future studies should consider measuring health-related language proficiency in addition to general proficiency. Thirdly, our study had a relatively limited scope on health-related behaviors and experiences of migrants. Navigating a foreign healthcare system generally requires additional skills [16, 54], such as understanding and negotiating the host country’s medical insurance and social welfare system. Future studies should consider measuring transcultural-transnational competencies related to health and social services in addition to language proficiency. Lastly, we did not measure several independent predictors of adherence to COVID-19 protective behaviors, including access to information about COVID-19 from the Ministry of Public Health (e.g., instructions, signs, and notifications in languages other than Thai), COVID-19 prevention guidelines and regulations at workplaces (e.g., the ’No mask = No entry’ sign in English or Burmese), overall awareness of COVID-19 related health information, and components of the health belief model (e.g., perceived susceptibility to COVID-19 infection, perceived severity of COVID-19 infection). These characteristics could have confounded the association between Thai language proficiency and adherence to COVID-19 protective behaviors. Future studies should consider measuring and controlling for these characteristics in a multivariable model, after which a more valid estimate of the association can be inferred.

Most of our participants reported following COVID-19 protective behaviors in the workplace and in the residence. The scores were relatively high, suggesting good adherence to protective behaviors. However, we collected our data in late 2022, approximately 2 years after the start of the pandemic. The findings of the study may not be generalizable to other contexts. Our questionnaires were anonymously self-administered, which reduced the potential for social desirability bias. However, self-serving bias might have existed among participants who deemed COVID-19 protective behaviors to be signals of good virtues. Furthermore, individual behaviors tend to change over time, and our study instrument did not capture these changes. Future studies should consider longitudinal data collection to provide more insights into the adoption of and adherence to protection behaviors [55–58].

In our study, workplace and residential COVID-19 prevention behaviors were not significantly associated with any domain of Thai language proficiency after adjusting for confounders. COVID-19 spread widely among Myanmar migrants in Thailand during the 2^nd^ wave of the COVID-19 outbreak in December 2020 [7], approximately two years before our survey. These past experiences might have induced a community-wide awareness and vigilance against COVID-19, irrespective of language proficiency, which might have contributed to the lack of associations found in our findings. A prior study in late 2021 found that Myanmar migrants in Southern Thailand were compliant with wearing face masks, hand washing, and avoiding unnecessary travel [10], similar to another study conducted in the Thai-Myanmar border areas [59].

The main contribution of this study to the literature was the measurement of self-reported Thai language proficiency using the CEFR framework, a globally recognized standard for language proficiency assessment. The instrument of this study can serve as the basis for future studies in both linguistics and public health, considering the extent to which language proficiency intertwines with social support and the ability to navigate the social, economic, and health systems in a foreign country [18–20]. However, a few limitations should be considered in the interpretation of the study findings. Firstly, we did not make a systematic content validity assessment of our questionnaire due to lack of time and resources and relied mainly on internal deliberations for questionnaire development. This lack of internal validity assessment should be considered as an important limitation in the interpretation of the study findings. Secondly, the lack of back-translation of the Thai language proficiency skills measurement questions poses a substantial threat to the validity of our exposure measurement. Thirdly, self-reporting of information did not preclude exposure and outcome measurements from social desirability and other types of biases. Fourthly, our questionnaire was available only in the Burmese language, whereas our participants included Myanmar migrants who might not have been able to read Burmese fluently. The potential language barrier could have similarly introduced information bias to our study findings. Fifthly, our data collection staff were ethnic Burmese, and pre-existing mistrust and social dynamics between ethnic groups could have also influenced and introduced bias to our study findings. Lastly, the findings of our study may be generalizable only to the context of the study areas, where migrants work mostly in factories, construction sites, and fisheries. Thai proficiency and behaviors of Myanmar migrants in predominantly service sector areas, such as at business establishments in big cities, could have been markedly different from those of our participants. Future studies should consider revising the measurement questions to reduce biases, inclusion of additional languages of Myanmar (e.g., Karen, Mon, Rakhine, etc.), and increase the diversity of the data collection staff.

## Conclusion

Myanmar migrants in low-paying occupations in Southern Thailand had generally low level of Thai language proficiency. However, language proficiencies were not associated with workplace and residential COVID-19 prevention behaviors after adjusting for confounders. Our study findings provide basic information that is potentially useful for stakeholders in migrant services, migrant health, and infectious disease control. However, information biases, language barriers among migrants who were not proficient in Burmese, and lack of generalizability should be considered as caveats in the interpretation of the study findings.

## Supporting information

S1 Table Response to Thai language proficiency assessment questions among participants. (PDF)

S2 Table Categories of Thai language proficiency among participants. (PDF)

## Abbreviations

CDC: Center for Disease Control
CEFR: Common European Framework Reference
CI: Confidence Interval
COVID-19: Coronavirus disease 2019
CPB: COVID-19 Protective Behavior
df: Degree of freedom
IQR: interquartile range
MPOH: Ministry of Public Health
NCD: Non-Communicable Disease
PIS: Participant Information Sheets
SD: Standard deviation
THB: Thai Baht
WHO: World Health Organization
