# Supplementary material for: Association between Thai language proficiency and adherence to COVID-19 protective behaviors (CPB) among Myanmar migrant workers in Southern Thailand
Source: PLoS One. 2024 Oct 25;19(10):e0312571. doi: 10.1371/journal.pone.0312571 (PMC11508075; doi:10.1371/journal.pone.0312571)
Supplement: S1 Table — (PDF) [file pone.0312571.s001.pdf]

Supplementary Table 1. Response to Thai Language Proficiency Assessment Questions among participants (n = 1050)

No	Language item	Frequency (%)		
		Cannot do	Partly do	Proficient
1.	Can you speak Thai in buying and bargaining?	560 (53.3%)	441 (42.0%)	49 (4.7%)
2.	Can you speak Thai in asking for the way/direction in the city?	620 (59.0%)	384 (36.6%)	46 (4.4%)
3.	Can you speak Thai in telling story to the police for help?	791 (75.3%)	222 (21.1%)	37 (3.5%)
4.	Can you read and understand Thai words/phrases on public signs?	1008 (96.0%)	39 (3.7%)	3 (0.3%)
5.	Can you read and understand Thai words and phrases in information leaflets or hospital appointment card?	1002 (95.4%)	43 (4.1%)	5 (0.5%)
6.	Can you read Thai newspapers headlines?	999 (95.1%)	46 (4.4%)	5 (0.5%)
7.	Can you listen and understand simple technical information or operating instruction at work in Thai language?	583 (55.5%)	428 (40.8%)	39 (3.7%)
8.	Can you listen and understand numbers, prices, times, and simple direction in Thai language?	585 (55.7%)	420 (40.0%)	45 (4.3%)
9.	Can you listen and understand Thai TV documentaries and movies	722 (68.8%)	296 (28.2%)	32 (3.0%)

10.	Can you fill in a questionnaire with your personal details (job, age, address) in Thai language?	985 (93.8%)	54 (5.1%)	11 (1.1%)
11.	Can you write short, simple notes and messages in Thai language?	999 (95.1%)	45 (4.3%)	6 (0.6%)
12.	Can you write a simple, short letter in Thai language?	1019 (97.0%)	27 (2.6%)	4 (0.4%)
