# Supplementary material for: Association between Thai language proficiency and adherence to COVID-19 protective behaviors (CPB) among Myanmar migrant workers in Southern Thailand
Source: PLoS One. 2024 Oct 25;19(10):e0312571. doi: 10.1371/journal.pone.0312571 (PMC11508075; doi:10.1371/journal.pone.0312571)
Supplement: S2 Table — (PDF) [file pone.0312571.s002.pdf]

Supplementary Table 2. Categories of Thai Language Proficiency (reading, writing, listening, speaking) among participants

A1 level	A2 level	B1 level		
		**Cannot do**	**Partly do**	**Proficient**
**Cannot do**	**Cannot do**	none	incoherent	incoherent
	**Partly do**	incoherent	incoherent	incoherent
	**Proficient**	incoherent	incoherent	incoherent
**Partly do**	**Cannot do**	< A1 level	< A1 level	A1 level
	**Partly do**	A1 level	A2 level	incoherent
	**Proficient**	incoherent	incoherent	incoherent
**Proficient**	**Cannot do**	A1 level	A1 level	incoherent
	**Partly do**	A1 level	A1 level	incoherent
	**Proficient**	A2 level	A2 level	B1 level
